# A hardware-efficient Berkeley gate for superconducting quantum processors

**DOI:** 10.1038/s41598-026-56236-8

**Published:** 2026-06-06

**Authors:** Muhammad AbuGhanem

**Affiliations:** Faculty of Science, Ain Shams University, Cairo, 11566 Egypt

**Keywords:** The Berkeley gate, Quantum process tomography, Hardware-efficient implementation, Experimental implementation of Berkeley gate, Superconducting quantum processors, NISQ era, Quantum benchmarking, Engineering, Mathematics and computing, Physics

## Abstract

The Berkeley gate is a high-performance, two-qubit entangling operation with particular potential for quantum error correction and fault-tolerant protocols. However, harnessing this potential on current noisy intermediate-scale quantum (NISQ) processors, requires efficient compilation and robust performance under realistic noise conditions. In this work, we demonstrate a hardware-efficient implementation of the Berkeley gate on a superconducting quantum processor. Using quantum process tomography (QPT), we experimentally characterize its performance and benchmark it against a noiseless quantum simulator to evaluate its practical reliability in the NISQ era. Experimental measurements confirm the gate’s correct logical action, producing the target partially entangled state with a subspace confinement probability of $P_{\text {succ}}^{\text {hardware}} \approx 95.96\%$ on real quantum hardware compared to 100% in quantum simulation. Results from QPT experiments show a simulated process fidelity of $\mathcal {F}_{\text {process}}^{\text {sim}} = 98.23\%$, while the experimental process fidelity on hardware is $\mathcal {F}_{\text {process}}^{\text {hardware}} = 91.76\%$. The observed discrepancy is analyzed in the context of device-specific noise sources, including qubit relaxation, dephasing, and state preparation and measurement (SPAM) errors. Our work provides a concrete fidelity benchmark for the Berkeley gate on superconducting hardware and quantify the impact of realistic noise on a non-trivial two-qubit operation, supporting its use in near-term algorithmic and error-correction applications.

## Introduction

Quantum information processing^1,2^ relies on high-fidelity two-qubit gates as fundamental building blocks for generating entanglement^3^, implementing algorithms^4^, and enabling error correction protocols^5,6^. Among the diverse set of two-qubit operations^2^, the Berkeley gate represents a distinct two-qubit entangling operation that occupies a strategic position in the space of universal quantum gates^7^. Unlike conventional gates such as the controlled-Z (CZ) or *i*SWAP^2^, the Berkeley gate offers an alternative entangling primitive that can be synthesized with minimal resource overhead^7^. Its structure places it in an advantageous region of the Weyl chamber, enabling efficient decomposition with reduced entangling cost compared to other gates of similar computational power^8,9^. This characteristic makes it particularly attractive for implementation on resource-constrained quantum architectures^10^.

Superconducting transmon qubits^11^ have established themselves as a leading platform for programmable quantum computation, offering increasingly sophisticated control over multi-qubit systems^1,6,12,13^. Within this framework, the evaluation of non-standard two-qubit gates like the Berkeley gate provides crucial insights into the flexibility and performance boundaries of contemporary quantum hardware^14^.

The expansion of available gate sets through efficient compilation has emerged as a key strategy for enhancing quantum algorithm performance on fixed-architecture processors^15^. Recent progress has demonstrated that non-native gates can achieve fidelities competitive with hardware-native operations^16^. In this work, we demonstrate a hardware-efficient implementation of the Berkeley gate on a superconducting quantum processor^14^. Experimental measurements confirm successful gate operation, with the output state predominantly occupying the expected computational subspace with 95.96% probability on quantum hardware, compared to 100% in quantum simulation, confirming correct logical operation with minimal state computational error despite device noise^10^.

Accurate characterization of such quantum operations demands rigorous benchmarking methodologies^17^. Quantum process tomography (QPT), serves as a comprehensive technique for reconstructing the complete process matrix of a quantum operation, enabling direct fidelity comparison with ideal gate performance^18–20^. By contrasting experimental implementations on real quantum hardware along with ideal quantum simulations, QPT elucidates the impact of device-specific noise channels and operational imperfections.

We employ full QPT to characterize the gate’s performance^18–20^, comparing results from physical hardware with ideal quantum simulations. Our tomography results reveal a process fidelity of 91.76% on hardware versus 98.23% in simulation. We analyze the fidelity reduction in the context of characterized device error mechanisms, providing insights into the practical limitations of complex two-qubit operations on current quantum hardware^10^. This study establishes a fidelity benchmark for the Berkeley gate and contributes to understanding the integration of advanced gate operations into near-term quantum algorithms^21^.

The Berkeley gate offers several theoretical advantages over conventional gates, including minimal two-qubit gate count for universal synthesis and efficient generation in certain physical platforms^7^. This work provides an experimental implementation and full quantum process tomography characterization of the Berkeley gate on a superconducting quantum processor, establishing a baseline process fidelity and quantifying the device-specific error mechanisms that limit its performance on current superconducting processors. Our results demonstrate that the Berkeley gate can be implemented with high fidelity using only two CNOT gates, and provide a quantitative benchmark of its performance under realistic noise conditions.

The structure of this paper is as follows. Section Background provides the theoretical background on the Berkeley gate, including its definition (The Berkeley Gate) and geometric characterization within the Weyl chamber (Geometric Representation in the Weyl Chamber). Section Methods details the experimental methods, encompassing the hardware-efficient decomposition (Hardware-Efficient Decomposition), direct measurement of the gate’s functionality and its susceptibility to state leakage (Direct State Measurements), and comprehensive QPT (Complete Gate Characterization), providing complete characterization of the implemented quantum process. Section Results and Discussion presents and discusses the results, analyzing the gate’s performance through direct state measurements,  full process tomography, and hardware performance analysis. Finally, Section Conclusion concludes by summarizing the findings and discussing their implications for near-term quantum computing.

## Background

Two-qubit gates serve as the fundamental entangling primitives in gate-based quantum computation^3^. While the CNOT gate represents the standard entangling operation in many quantum algorithms and error correction codes^4,5^, various hardware platforms implement different native interactions. For superconducting quantum processors^1^, common entangling gates include the echoed cross-resonance gate (ECR)^15,22,23^, which serves as the native interaction on most IBM Quantum’s quantum systems^23^, as well as the *i*SWAP and $\sqrt{i\text {SWAP}}$ gates frequently employed in tunable-coupler architectures^12,24,25^. Among these alternatives, the Berkeley gate emerges as a significant two-qubit operation that exhibits particular advantages for hardware-efficient implementation on superconducting qubit systems^7^.

### The Berkeley gate

The Berkeley gate (denoted as B) is a two-qubit entangling operation that has been proposed for superconducting qubits^7^. It serves as a universal entangling primitive, meaning that when combined with arbitrary single-qubit rotations, it can construct any two-qubit unitary.

Beyond this universality, the Berkeley gate offers several specific advantages over conventional gates such as CNOT. First, as originally shown in Ref^7^, an arbitrary two-qubit unitary can be realized using only two applications of the B gate together with single-qubit rotations. The B gate therefore achieves the minimum possible two-qubit gate count for universal two-qubit synthesis.

Second, in physical implementations where the underlying Hamiltonian is of the form in Eqn. (1), the B gate can be generated directly via free evolution under this Hamiltonian, requiring less application time than a CNOT compiled from the same interaction^7^. These properties make the B gate not merely a distinct alternative to CNOT, but a potentially more efficient primitive for certain tasks and platforms.

The Berkeley gate derives from an exchange-type interaction described by the Hamiltonian:

1 $$\begin{aligned} \mathcal {H} = 2X \otimes X + Y \otimes Y, \end{aligned}$$

where $X$ and $Y$ are the standard Pauli operators.

$$X = \begin{pmatrix} 0 & 1\\ 1 & 0 \end{pmatrix} , \quad Y = \begin{pmatrix} 0 & -i\\ i & 0 \end{pmatrix}.$$

The time evolution generated by this Hamiltonian over a duration $t = \pi /8$ yields the Berkeley gate:

2 $$\begin{aligned} U^\text {B} = \exp \Big (i \frac{\pi }{8}\,(2X\otimes X + Y\otimes Y)\Big ). \end{aligned}$$

This formulation reveals the gate’s physical interpretation as a controlled exchange interaction with specific weighting between the *XX* and *YY* coupling components.

Expressed in the computational basis ${|00\rangle ,|01\rangle ,|10\rangle ,|11\rangle }$, the unitary matrix for the Berkeley gate is:

3 $$\begin{aligned} U^\text {B}= \frac{\sqrt{2-\sqrt{2}}}{2}\begin{pmatrix} 1+\sqrt{2}& 0& 0& i\\ 0& 1& i(1+\sqrt{2})& 0\\ 0& i(1+\sqrt{2})& 1& 0\\ i& 0& 0& 1+\sqrt{2}\\ \end{pmatrix} \end{aligned}$$

While this work focuses on superconducting qubits, the Hamiltonian in Eqn. (1), represents a general anisotropic exchange interaction that is not specific to this quantum architecture^26,27^. Its realization in other quantum hardware is possible where suitable coupling mechanisms can be engineered. This makes the Berkeley gate of broader relevance to quantum information processing.

These advantages–minimal two-qubit gate count, direct Hamiltonian generation, and full universality–make the Berkeley gate a compelling candidate for efficient quantum circuit synthesis. The reduced circuit depth lowers susceptibility to decoherence and gate errors, offering practical benefits for algorithm realization on NISQ processors. 

### Geometric representation in the Weyl chamber

**Fig. 1:**
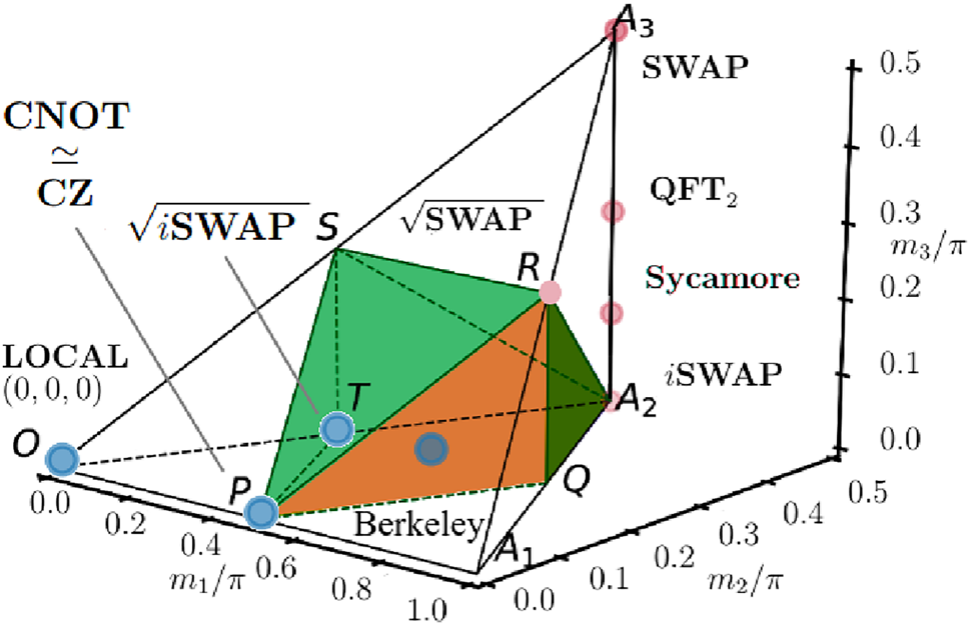
Geometric representation of two-qubit gates in the Weyl chamber, highlighting the position of the Berkeley gate. The chamber illustrates the local equivalence classes of two-qubit gates, parameterized by the canonical triple $(m_1,m_2,m_3)$. The Berkeley gate is located at $(\pi /4,\pi /8,0)$, lying on the edge between the CNOT class $(\pi /4,0,0)$ and the iSWAP class $(\pi /4,\pi /4,0)$. This placement identifies the Berkeley gate as a perfect entangler, intermediate in character between CNOT- and iSWAP-type interactions. The figure also highlights standard reference points such as the identity $(0,0,0)$, SWAP $(\pi /4,\pi /4,\pi /4)$, and controlled-phase gates, providing a geometric context for the Berkeley gate’s entangling capabilities.

The classification of two-qubit gates is often studied through their representation in the Weyl chamber, a geometric framework that captures the local equivalence classes of two-qubit unitaries^8,9^. In this representation, gates that differ only by single-qubit rotations occupy the same equivalence class and can be positioned within a tetrahedral region defined by the chamber coordinates.

The Berkeley gate occupies a distinct point within the Weyl chamber^8,9^, making it inequivalent under local operations to more conventional gates such as CNOT or CZ^2,9^. This unique positioning underscores its role as a distinct entangling primitive and motivates experimental investigation of its performance on realistic hardware. Analysis within the Weyl chamber framework provides a systematic approach for comparing entangling power and resource requirements across different two-qubit gate families.

For the Berkeley gate $U^\text {B}$ (Eq. (2)), the canonical decomposition^8,9^ yields the coordinates $(\pi /4, \pi /8, 0)$. This placement positions the Berkeley gate along the edge connecting the CNOT class $(\pi /4,0,0)$ and the iSWAP class $(\pi /4,\pi /4,0)$. The gate’s location confirms its status as a perfect entangler and reveals its intermediate character between CNOT-type and iSWAP-type interactions (Fig. 1). This geometric characterization provides a compact description of the gate’s entangling capabilities and underlines its experimental relevance for superconducting architectures, where such hybrid interactions arise naturally.

## Methods

**Fig. 2:**
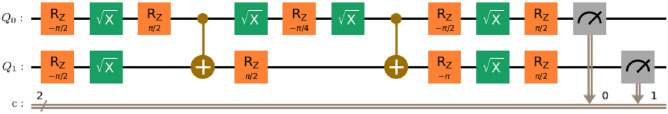
Hardware-efficient quantum circuit implementation of the Berkeley gate. The decomposition utilizes single-qubit gates (including $\sqrt{X}$ and $R_Z$ rotations) and only two CNOT gates, minimizing circuit depth for execution on superconducting quantum processors. The specific sequence of operations ensures faithful implementation of the Berkeley gate while maintaining compatibility with device connectivity constraints. This implementation significantly reduces the circuit’s vulnerability to decoherence and two-qubit gate errors compared to generic two-qubit gate implementations, that might require three or more CNOT gates.

### Hardware-efficient decomposition

The implementation of the Berkeley gate on contemporary superconducting quantum processors^1^ requires decomposition into the device’s native gate set. For the quantum hardware used in this implementation (ibm_nairobi), the native gate set consists of $R_Z(\theta )$, $\sqrt{X}$, *X*, and CNOT. We developed a hardware-efficient decomposition that expresses the Berkeley gate using only two CNOT gates.

The decomposition, shown in Fig. 2, combines single-qubit $R_Z(\theta )$ rotations, $\sqrt{X}$ gates, and exactly two CNOT gates. The decomposition is platform-agnostic and can be executed on any quantum processor supporting CNOT and the required single-qubit rotations. For our experimental implementation, we selected a directly coupled qubit pair ($Q_0$ and $Q_1$, which are physically adjacent on the ibm_nairobi coupling map). We emphasize that no device-specific pulse-level or calibration-aware optimization was performed; the circuit is executed using the standard native gates of the target hardware.

By comparison, the canonical Kraus-Cirac decomposition for an arbitrary two-qubit unitary requires up to three CNOTs^28,29^. This reduction in entangling gate count directly reduces circuit depth and susceptibility to decoherence and two-qubit gate errors that dominate in NISQ-era devices^10^. The resulting hardware-aware decomposition positions the Berkeley gate as a practical and high-performance entangling operation for near-term quantum algorithms.

We characterized the Berkeley gate using a comparative approach that executed identical circuits on both a noiseless quantum simulator and physical quantum hardware. This methodology isolates hardware-induced errors from imperfections inherent to the gate decomposition itself.

### Direct state measurements

The functionality of the proposed Berkeley gate implementation was assessed by applying its circuit to the input state $\left| {0}\right\rangle \otimes \left| {0}\right\rangle$. The resulting output distribution across the computational basis states was measured using 13,000 shots. This test provides an operational measure of the gate’s functionality and its susceptibility to computational errors manifesting as population in the off-target states.

To establish a performance baseline, we first executed the proposed circuit on a noiseless quantum simulator (qasm_simulator). This idealized environment provides a reference for the gate’s behavior in the absence of decoherence, control errors, and measurement imperfections. All simulations employed identical circuit configurations and measurement protocols as those used on physical hardware.

Then we perform direct state measurement on physical hardware. The proposed circuit was executed with the input state $\left| {00}\right\rangle$ to verify its functional operation. The ideal output produces the partially entangled state $\cos (\pi /8)|00\rangle + i\sin (\pi /8)|11\rangle$. This test used 13,000 shots to obtain statistically significant measurement distributions. The key metric extracted is the subspace success probability $P_{\text {succ}} = p_{\left| {00}\right\rangle } + p_{\left| {11}\right\rangle }$ from the direct Berkeley circuit execution.

**Fig. 3:**
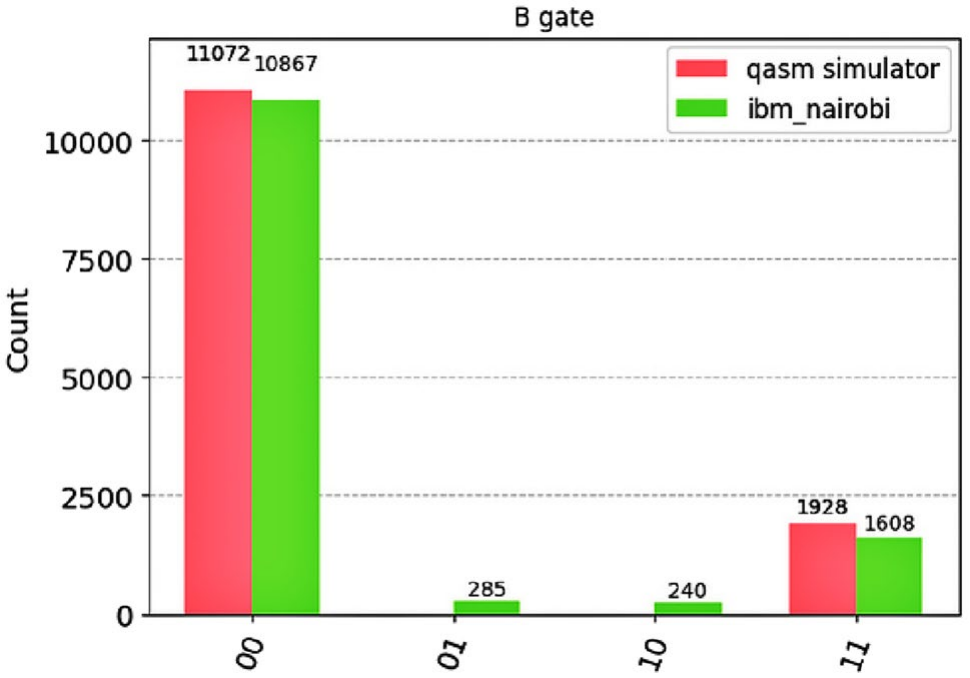
Measurement outcomes for the Berkeley gate circuit implementation with input state $|00\rangle$. The circuit was compiled to match the connectivity constraints of the target quantum processor. Results compare quantum simulation (red) and hardware execution on ibm_nairobi (green) from 13,000 shots each. The simulator results (85.2% in $|00\rangle$, 14.8% in $|11\rangle$) closely match the theoretical prediction of 85.4% and 14.6% for the partially entangled target state. The hardware results (83.6% in $|00\rangle$, 12.4% in $|11\rangle$, 4.0% distributed across $|01\rangle$ and $|10\rangle$) demonstrate successful gate operation with minimal state computational error. The corresponding subspace confinement probabilities are $P_{\text {succ}}^{\text {sim}}=100.0\%$ and $P_{\text {succ}}^{\text {exp}}=95.96\%$. The actual process fidelity obtained from quantum process tomography ($\mathcal {F}_{\text {process}}^{\text {sim}}=98.2\%$ and $\mathcal {F}_{\text {process}}^{\text {exp}}=91.8\%$) confirms preservation of quantum coherence beyond what population statistics alone can capture.

### Complete gate characterization

The performance of the Berkeley gate implementation was rigorously evaluated through full QPT, providing complete characterization of the implemented quantum process. This comprehensive protocol enables reconstruction of the full process matrix and direct fidelity comparison with the ideal gate operation^30,31^.

The QPT procedure involved preparing the complete set of input states from the Pauli basis $\{I, X, Y, Z\}^{\otimes 2}$ and performing measurements in all necessary bases to reconstruct the process matrix. In this work, the QPT procedure follows the standard Choi-matrix reconstruction method. A tomographically complete set of input states is prepared from the eigenstates of the Pauli operators. For each input state, the output state is reconstructed via full state tomography using measurements in the Pauli bases. Then, the process matrix $\chi$is reconstructed. For a comprehensive description of standard quantum process tomography, we refer to Refs^18–20^. The Choi-matrix reconstruction procedure is detailed in Ref^31^, which provides a full implementation of Choi-based QPT for superconducting architectures.

Experimental data were collected using 4,000 shots per measurement setting to ensure statistical significance. The same procedure was executed in parallel on a quantum simulator (qasm_simulator) to establish a theoretical baseline and on the IBM Quantum ibm_nairobi superconducting processor to assess performance under realistic noise conditions.

The primary metric extracted from this characterization is the process fidelity $\mathcal {F}_{\text {process}}$, which quantifies the overlap between the experimentally implemented process and the ideal Berkeley gate operation^32,33^. This metric provides a rigorous benchmark of gate implementation quality, with unity fidelity representing perfect execution.

## Results and discussion

**Fig. 4:**
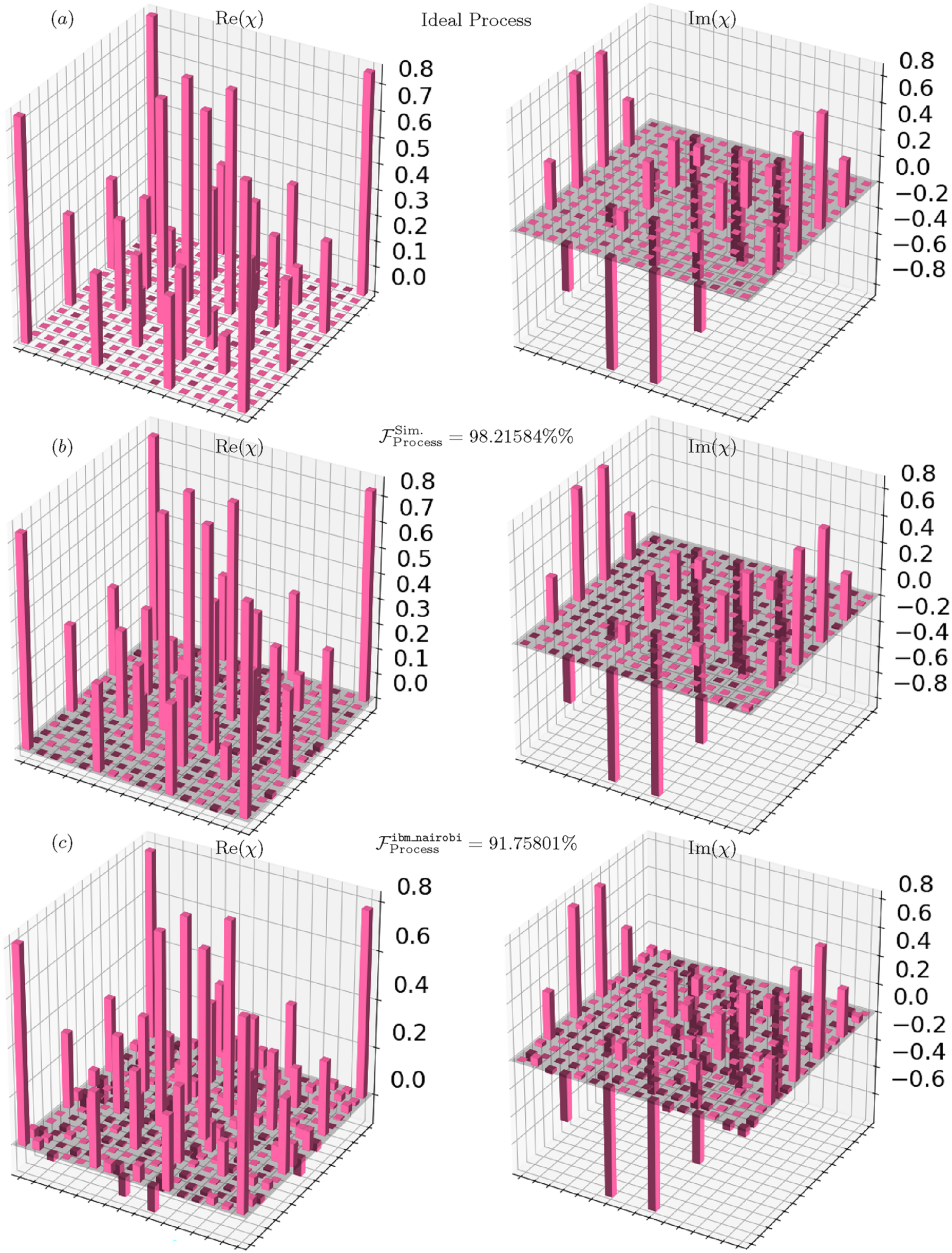
Quantum process tomography of the Berkeley gate. (a) Ideal theoretical process ($\mathcal {F}_{\text {process}}^{\text {Ideal}} = 100\%$). (b) Process reconstructed from data from the quantum simulator, showing a process fidelity of ($\mathcal {F}_{\text {process}}^{\text {qasm}} =98.21584\%$. (c) Process reconstructed from data from the physical hardware, showing a process fidelity of ($\mathcal {F}_{\text {process}}^{\text {hardware}} =91.75801\%$. The visual degradation in the experimental process reflects the impact of device noise on gate performance.

**Fig. 5:**
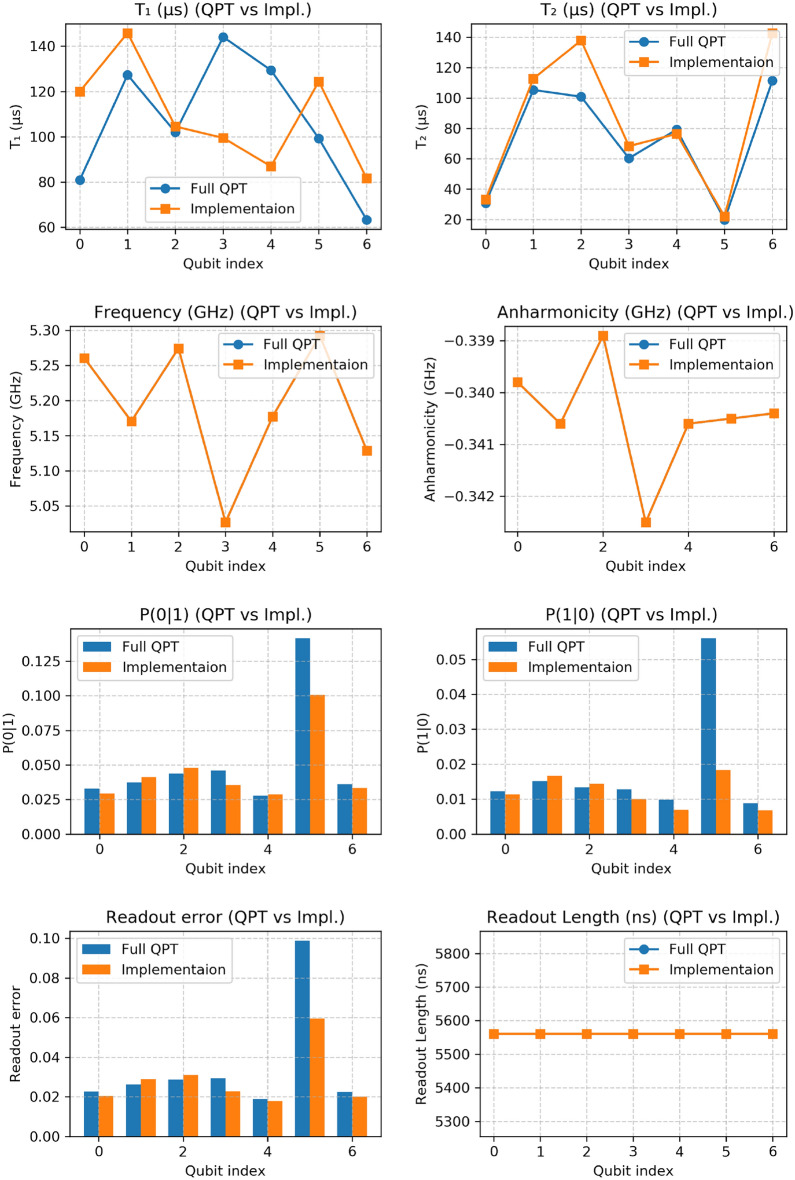
Qubit-specific metrics for the quantum device, ibm_nairobi, during both QPT experiments and the real execution of Berkeley gate. The two qubits used in our implementation ($Q_0$ and $Q_1$) are directly coupled adjacent qubits. Each subplot presents a side-by-side comparison of a specific parameter: $T_1$ relaxation time, $T_2$ dephasing time, qubit frequency, anharmonicity, probability of measuring $|0\rangle$ when $|1\rangle$ was prepared, probability of measuring $|1\rangle$ when $|0\rangle$ was prepared, readout length, and readout error. These parameters directly influence gate fidelity, SPAM errors, and coherence-limited performance. The consistency and variation of these parameters across different qubits provide insights into the stability and performance of the quantum device.

### Hardware implementation efficiency

The computational-basis measurement statistics provide a direct assessment of the Berkeley gate’s operational performance. When applied to the input state $|00\rangle$, the ideal gate prepares the partially entangled state $\cos (\pi /8)|00\rangle + i\sin (\pi /8)|11\rangle$, corresponding to theoretical populations of approximately 85.4% for $|00\rangle$ and 14.6% for $|11\rangle$.

Experimental results closely match this prediction. On the qasm simulator (13,000 shots), measurements yielded populations of $|00\rangle$ (85.2%) and $|11\rangle$ (14.8%), with no population in the $|01\rangle$ and $|10\rangle$ states. The quantum hardware implementation produced a similar distribution: $|00\rangle$ (83.6%), $|11\rangle$ (12.4%), with a total of 4.04% population incorrectly appearing in $|01\rangle$ (2.2%) and $|10\rangle$ (1.8%).

We quantify the gate’s ability to confine the output to the expected subspace with the success probability:

$$P_{\text {succ}} = p_{\left| {00}\right\rangle } + p_{\left| {11}\right\rangle }.$$

Defining the success probability as the likelihood of observing the expected outcomes $|00\rangle$ or $|11\rangle$, we obtain

$$P_{\text {succ}}^{\text {sim}} = 100.0\%, \qquad P_{\text {succ}}^{\text {exp}} \approx 95.96\%.$$

Population in the states $|01\rangle$ and $|10\rangle$ thus accounts for approximately $4\%$ of the outcomes on hardware. This yields $P_{\text {succ}}^{\text {sim}} = 100.0\%$ for the simulator and $P_{\text {succ}}^{\text {exp}} = 95.96\%$ for the hardware. The close agreement between the simulated populations (85.2%/14.8%) and the theoretical prediction (85.4%/14.6%) validates the correctness of the gate decomposition. The slight additional population shift towards $|00\rangle$ on hardware is consistent with expected noise effects such as amplitude damping ($T_1$ relaxation).

This high success probability demonstrates that the Berkeley gate reliably performs its intended logical function on current hardware, with the output state predominantly confined to the target subspace $\{|00\rangle , |11\rangle \}$ and minimal population in the undesired computational states $|01\rangle$ and $|10\rangle$.

It should be noted that the state preparation fidelity reported here is specific to the $\left| {00}\right\rangle$ input state. While such a measurement provides a useful functional check, a comprehensive benchmark across all two-qubit basis states ($\left| {00}\right\rangle , \left| {01}\right\rangle , \left| {10}\right\rangle , \left| {11}\right\rangle$) would be required to fully characterize input-state-dependent performance. The complete validation of the Berkeley gate implementation is instead provided by the quantum process tomography results presented in Section Experimental Quantum Process Fidelity, which reconstruct the full quantum process and constitute the primary benchmark for gate correctness.

### Experimental quantum process fidelity

Beyond the population statistics presented above, QPT provides a complete characterization of the implemented quantum operation, including crucial phase information. The performance of the Berkeley gate implementation was quantitatively assessed through QPT, with results summarized in Fig. 4. The reconstructed process matrices provide a visual representation of the gate’s behavior across different implementation environments.

The noise-free simulation on the qasm_simulator yielded a process fidelity of:

$$\mathcal {F}_{\text {process}}^{\text {sim}} = 0.9822 \quad (98.23\%).$$

This high fidelity value validates the correctness of our gate decomposition and confirms that the theoretical implementation accurately reproduces the ideal Berkeley gate operation. The minor deviation from unity fidelity is attributable to finite-sampling effects inherent in the tomographic reconstruction process.

Execution on the ibm_nairobi superconducting processor resulted in a measured process fidelity of:

$$\mathcal {F}_{\text {process}}^{\text {exp}} = 0.9176 \quad (91.76\%).$$

Table 1 summarizes these key performance metrics alongside the subspace success probabilities from Section Hardware Implementation Efficiency. The hardware-induced degradation of 6.47% in process fidelity and 4.04% in subspace success probability consistently reflects the impact of device noise on gate performance.

The observed process fidelity reduction of approximately 6.5 percentage points between simulation and hardware implementation represents the cumulative effect of device-specific noise processes^10^. Despite this reduction, the experimental process fidelity remains above 90%, demonstrating the feasibility of implementing non-standard two-qubit gates with reasonably high accuracy on current NISQ-era hardware.

This fidelity reduction manifests visually in the process matrices (Fig. 4), which reveal the specific ways in which hardware imperfections distort the ideal gate operation. The experimental matrix maintains the overall structure of the ideal Berkeley gate but exhibits increased off-diagonal elements and reduced contrast in key matrix components, consistent with the effects of decoherence and control errors (Fig. 5).

Standard QPT is inherently sensitive to state preparation and measurement (SPAM) errors. The reported experimental process fidelity of 91.76% therefore includes these SPAM contributions and should be interpreted as a conservative lower bound on the true gate fidelity under the given experimental conditions. SPAM-robust techniques such as interleaved randomized benchmarking^34^ or cycle benchmarking^35^ would be required to obtain a SPAM-corrected fidelity estimate; we identify this as a valuable direction for future characterization of the Berkeley gate on superconducting quantum processors.

Nonetheless, the preservation of high fidelity in the hardware implementation–even under this conservative interpretation–underscores the effectiveness of our hardware-efficient decomposition strategy. By minimizing circuit depth and leveraging optimized gate sequences, we mitigate the impact of noise sources that typically dominate in superconducting quantum processors. This result positions the Berkeley gate as a viable entangling primitive for near-term quantum applications requiring alternative two-qubit interactions.

**Table 1:** Summary of Berkeley gate performance metrics. Quantum process tomography fidelities ($\mathcal {F}_{\text {process}}$) are obtained from 4,000 shots per measurement setting; subspace success probabilities ($P_{\text {succ}} = p_{\left| {00}\right\rangle } + p_{\left| {11}\right\rangle }$) are obtained from 13,000 shots with input state $\left| {00}\right\rangle$. The hardware-induced degradation ($\Delta$) is shown for both metrics.

**Gate**	Process Fidelity$\mathcal {F}_{\text {process}}$		$\Delta \mathcal {F}_{\text {process}}$ **(Hardware Drop)**	Subspace Success$P_{\text {succ}}$		$\Delta P_{\text {succ}}$ (Hardware Drop)
	**Simulator**	**Hardware**		**Simulator**	**Hardware**	
Berkeley	98.23%	91.76%	6.47%	100.00%	95.96%	4.04%

**Table 2:** Specification metrics for qubits used in the Berkeley gate experiment on the ibm_nairobi quantum computer. Parameters include relaxation times ($T_1$), dephasing times ($T_2$), frequencies, anharmonicities, readout errors, state preparation and measurement (SPAM) errors (or conditional measurement probabilities P(0|1) and P(1|0)), and readout durations.

Parameter	$Q_0$	$Q_1$	$Q_2$	$Q_3$	$Q_4$	$Q_5$	$Q_6$
$T_1$ ($\mu$s)	119.97	145.91	104.49	99.46	86.87	124.40	81.50
$T_2$ ($\mu$s)	33.18	112.72	137.78	68.28	76.34	22.01	142.77
Frequency (GHz)	5.2605	5.1704	5.2743	5.0267	5.1772	5.2925	5.1287
Anharmonicity (GHz)	−0.3398	−0.3406	−0.3389	−0.3425	−0.3406	−0.3405	−0.3404
Readout Error	0.0204	0.0288	0.0311	0.0228	0.0179	0.0595	0.0201
P(0|1)	0.0294	0.0410	0.0478	0.0356	0.0288	0.1006	0.0334
P(1|0)	0.0114	0.0166	0.0144	0.0100	0.0070	0.0184	0.0068
Readout Len (ns)	5560.89	5560.89	5560.89	5560.89	5560.89	5560.89	5560.89

Qubits $Q_0$ and $Q_1$ were utilized for implementing the Berkeley gate.

**Table 3:** Characterization of the 7-qubit ibm_nairobi processor during quantum process tomography experiments.

Parameter	$Q_0$	$Q_1$	$Q_2$	$Q_3$	$Q_4$	$Q_5$	$Q_6$
$T_1$ ($\mu$s)	80.78	127.35	101.96	144.12	129.40	99.23	63.21
$T_2$ ($\mu$s)	30.70	105.23	100.78	60.27	79.24	19.78	111.29
Frequency (GHz)	5.2605	5.1704	5.2743	5.0267	5.1772	5.2925	5.1287
Anharmonicity (GHz)	−0.3398	−0.3406	−0.3389	−0.3425	−0.3406	−0.3405	−0.3404
Readout Error	0.0225	0.0263	0.0286	0.0294	0.0188	0.0987	0.0224
P(0|1)	0.0328	0.0374	0.0438	0.0460	0.0278	0.1414	0.0360
P(1|0)	0.0122	0.0152	0.0134	0.0128	0.0098	0.0560	0.0088
Readout Length (ns)	5560.89	5560.89	5560.89	5560.89	5560.89	5560.89	5560.89

### Analysis of hardware performance

All experiments were performed on the ibm_nairobi quantum computer, a seven-qubit device based on IBM’s Falcon superconducting architecture. The processor employs fixed-frequency transmon qubits, with bidirectional couplings between physically adjacent qubits. The complete coupling connectivity is: $Q_0 \leftrightarrow Q_1,\quad Q_1 \leftrightarrow Q_2,\quad Q_1 \leftrightarrow Q_3,\quad Q_3 \leftrightarrow Q_5,\quad Q_4 \leftrightarrow Q_5,\quad Q_5 \leftrightarrow Q_6,$ where bidirectional coupling is indicated by $\leftrightarrow$. Our experiments specifically used the directly connected qubit pair $Q_0-Q_1$. The device has a Quantum Volume of 32 and its native gate set consists of $\{\text {ID}, R_Z(\theta ), \sqrt{X}, X, \text {CNOT}\}$. Calibration data for the qubits used in this work–including coherence times ($T_1$, $T_2$), readout errors, and gate errors–are provided in Tables 2 and 3 and visualized in Fig. 5. These parameters are discussed below in the context of our observed fidelity degradation.

The observed performance reduction between simulated ($\mathcal {F}_{\text {process}}^{\text {sim}} = 0.982$) and experimental ($\mathcal {F}_{\text {process}}^{\text {exp}} = 0.918$) implementations can be systematically attributed to specific noise mechanisms in superconducting processors. Analysis of device calibration data (Table 2 for circuit execution; Table 3 for QPT) reveals several contributing factors:

1. Coherence limitations: The selected qubits ($Q_0$ and $Q_1$) exhibited coherence times of $T_1^{Q_0} = 80.78\ \mu$s, $T_2^{Q_0} = 30.70\ \mu$s and $T_1^{Q_1} = 127.35\ \mu$s, $T_2^{Q_1} = 105.23\ \mu$s. The finite execution time of the decomposed circuit permits significant decoherence.
2. Two-qubit gate errors: The decomposition requires two CNOT gates, which typically exhibit 1–3% infidelity per gate on superconducting processors. As the dominant error source, their cumulative effect substantially contributes to the observed fidelity reduction.
3. SPAM errors: Readout errors for $Q_0$ and $Q_1$ were measured at 2.25% and 2.63%, with conditional probabilities $\text {P(0|1)} \approx 3.7\%$ and $\text {P(1|0)} \approx 1.5\%$. These errors corrupt the tomographic data, introducing systematic biases in fidelity estimation^17^.

The measurement outcomes provide additional evidence, while simulation shows near-perfect confinement to expected states, hardware exhibits 4.04% population into $|01\rangle$ and $|10\rangle$, consistent with decoherence and gate errors.

Despite these challenges, the achieved experimental fidelity of 91.76% demonstrates the effectiveness of our hardware-efficient decomposition. By minimizing circuit depth to only two CNOT gates, we reduce vulnerability to accumulated errors compared to implementations requiring three or more entangling operations. This performance is competitive with standard two-qubit gate benchmarks on similar NISQ devices^30^ and confirms the practical viability of the Berkeley gate for near-term quantum applications.

The results highlight the critical importance of co-design approaches–where gate decomposition strategies are optimized specifically for target hardware characteristics—for maximizing performance on current quantum processors. The Berkeley gate’s efficient implementability, combined with its theoretical advantages as an entangling primitive, positions it as a valuable component in the toolbox of quantum circuit compilation techniques.

## Conclusion

We have demonstrated an experimental implementation and characterization of the Berkeley gate on a superconducting quantum processor, achieving an experimental process fidelity of $\mathcal {F}_{\text {process}} = 0.918$ through comprehensive QPT. This performance, which represents a $6.5\%$ reduction from the quantum simulated fidelity of $\mathcal {F}_{\text {process}} = 0.982$, highlights both the capabilities and limitations of current NISQ-era hardware for implementing non-standard two-qubit operations.

The success of our implementation stems from the Berkeley gate’s favorable decomposition properties, requiring only two CNOT gates compared to the three or more typically needed for arbitrary two-qubit unitaries. This minimal-depth circuit design significantly mitigates error accumulation from decoherence and gate imperfections, as evidenced by the high operational success probability of $P_{\text {succ}} = 95.96\%$ observed in computational basis measurements. The gate’s efficient implementability, combined with its theoretical position in the Weyl chamber as an intermediate entangling primitive between CNOT and iSWAP-type gates, makes it particularly valuable for quantum circuit compilation and algorithm design where alternative entangling interactions are beneficial.

Our error analysis reveals that the fidelity reduction is consistent with characterized device limitations, including finite coherence times ($T_1$, $T_2$), CNOT gate errors, and SPAM imperfections. The observed population into undesired computational states ($4.04\%$) and the additional shift in populations beyond the expected theoretical ratio further corroborate the impact of these noise mechanisms. Nevertheless, the preservation of high fidelity under realistic noise conditions confirms the robustness of our decomposition strategy.

Looking forward, several pathways exist for enhancing Berkeley gate performance on superconducting hardware. Pulse-level optimizations could potentially reduce gate times and minimize susceptibility to decoherence. Dynamical decoupling sequences may help suppress environmental noise during gate execution, while advanced error mitigation techniques could further improve fidelity estimates. Additionally, the Berkeley gate’s efficient decomposition makes it a promising candidate for integration into larger quantum circuits, particularly in applications requiring alternative entangling interactions beyond standard CNOT gates.

This work establishes the Berkeley gate as a practical and efficient two-qubit primitive for near-term quantum computing. By providing a comprehensive fidelity benchmark and detailed performance analysis, we contribute to the growing toolkit of quantum operations available for algorithm development on NISQ devices. As quantum hardware continues to advance, the co-design approach demonstrated here–where gate selection and decomposition are optimized for specific hardware characteristics–will remain essential for maximizing computational capabilities on superconducting quantum processors.

## Data Availability

All data generated or analyzed during this study are included in this published article.
